# Association of Circulating Magnesium Levels in Patients With Alzheimer's Disease From 1991 to 2021: A Systematic Review and Meta-Analysis

**DOI:** 10.3389/fnagi.2021.799824

**Published:** 2022-01-10

**Authors:** Ke Du, Xi Zheng, Zi-Tai Ma, Jun-Ya Lv, Wen-Juan Jiang, Ming-Yan Liu

**Affiliations:** ^1^Department of Pharmacology, School of Pharmacy, China Medical University, Shenyang, China; ^2^Department of Geriatrics, The First Affiliated Hospital of China Medical University, Shenyang, China

**Keywords:** magnesium, serum, plasma, CSF, Alzheimer's disease, meta-analysis

## Abstract

Alzheimer's disease (AD) remains a medical and social challenge worldwide. Magnesium (Mg) is one of the most frequently evaluated essential minerals with diverse biological functions in human body. However, the association between circulating Mg levels and AD remains controversial. We conducted a meta-analysis of 21 studies published between 1991 and 2021 to determine whether the Mg levels in the blood and cerebrospinal fluid (CSF) are abnormal in AD. Literatures were searched in PubMed, Web of Science, China National Knowledge Infrastructure (CNKI), and Wanfang Data without language limitations. A pooled subject sample including 1,112 AD patients and 1,001 healthy controls (HCs) was available to assess Mg levels in serum and plasma; 284 AD patients and 117 HCs were included for Mg levels in CSF. It was found that serum and plasma levels of Mg were significantly reduced in AD patients compared with HCs (standardized mean difference [SMD] = −0.89; 95% confidence interval [CI] [−1.36, −0.43]; *P* = 0.000). There was statistically non-significant for Mg level in CSF between AD and HCs, whereas a decreased tendency were detected (SMD = −0.16; 95% CI [−0.50, 0.18]; *P* = 0.364). .In addition, when we analyzed the Mg levels of serum, plasma and CSF together, the circulating Mg levels in AD patients was significantly lower (SMD = −0.74, 95% CI [−1.13; −0.35]; *P* = 0.000). These results indicate that Mg deficiency may be a risk factor of AD and Mg supplementation may be a potentially valuable adjunctive treatment for AD.

**Systematic Review Registration:** www.crd.york.ac.uk/PROSPERO/, registration number CRD42021254557.

## Introduction

Alzheimer's disease (AD) is the most common cause of dementia, typified by cognitive impairment and brain lesions. The typical pathological changes include plaques formed by beta-amyloid (Aβ) aggregation and intracellular neurofibrillary tangles, as well as prolonged inflammation (Akiyama et al., 2000; Lesne et al., 2006). Although there are many basic and clinical researches on AD, the etiology of AD has not been comprehensively elucidated. Currently, AD treatment largely depends on cholinesterase inhibitors, which is only a symptomatic therapy. It means that these drugs have limited efficacy on AD progression (Sharma, 2019). Therefore, it is necessary to evaluate the risk factors for AD to provide an opportunity for delaying the AD progression.

Notably, the dyshomeostasis of nutritional minerals has been associated with AD progression (Gonzalez-Dominguez et al., 2014). Previous studies have proposed the imbalance of several minerals, such as zinc (Ventriglia et al., 2015; Kawahara et al., 2018), iron (Belaidi and Bush, 2016; Lane et al., 2018), copper (Donnelly et al., 2007; Sensi et al., 2018), and manganese (Du et al., 2017; Mezzaroba et al., 2019), as risk factors in AD. Magnesium (Mg) is an essential for the maintenance of human health. Mg plays a critical role in nerve transmission and neuromuscular conduction in nervous system and has a protective effect against excitotoxicity inducing neuronal death (Kirkland et al., 2018). It is associated with multiple neurological disorders in central nervous system, including migraine (Chiu et al., 2016; Dolati et al., 2020), epilepsy (Abdullahi et al., 2019; Yary and Kauhanen, 2019) and Parkinson's disease (Oyanagi and Hashimoto, 2011; Shen et al., 2019). Recently, Mg investigations are paid more attention in AD researchers. However, contradictory results exist regarding abnormal Mg levels in AD patients. Several reports have described the systemic levels of Mg were significantly reduced in AD (Lemke, 1995; Kurup and Kurup, 2003; Cilliler et al., 2007; Vural et al., 2010; Barbagallo et al., 2011; Singh et al., 2014; Ahmed et al., 2017; Balmus et al., 2017), but others have reported no differences or even elevated Mg levels in AD patients (Zhu et al., 1997; Cheng et al., 1999; Alimonti et al., 2007; Liu, 2008; Bostrom et al., 2009a,b; Gustaw-Rothenberg et al., 2010; Hozumi et al., 2011; Koc et al., 2015; Wang, 2015; Zheng, 2015; Xu et al., 2018; Jouini et al., 2021). However, these studies only involved single case-control investigations with small sample size. Therefore, they may lack sufficient power, leading to limitation the scope of their findings.

Here, we conducted a systematic review to comprehensively estimate variations in circulating Mg levels (in the plasma, serum, and cerebrospinal fluid [CSF]) in AD patients compared with healthy controls (HCs). The aim of the present study was to gain additional insights into maintaining an adequate nutritional state for AD prevention or treatment.

## Methods

### Search Strategies and Selection of Studies

The review was conducted in accordance with the “Preferred Reporting Items for Systematic reviews and Meta-Analyses” (PRISMA) statement (Moher et al., 2010) and was registered in PROSPERO (CRD42021254557). We searched relevant literature from PubMed, Web of Science, China National Knowledge Infrastructure (CNKI), and WANGFANG, selecting studies from 1991 to 2021. The search terms were “magnesium,” “Alzheimer's disease,” “serum,” “plasma,” or “CSF.” Both English and Chinese languages were used. The Supplementary Materials present the PRISMA checklist (**Supplementary PRISMA Checklist**) and detailed search strategy (Supplementary Methods: **Search strategy**). The inclusion criteria were: (1) case-control study design; (2) human subjects; (3) both AD and control groups described in terms of sample size and Mg concentration in serum, blood, plasma, or CSF. The exclusion criteria were: (1) letter, review, or case reports; (2) duplicated studies with repeated data; (3) *in vitro* or laboratory studies; (4) animal studies; (5) studies lacking quantitative data on Mg concentrations.

### Extraction of Data and Quality Evaluation

The studies were evaluated separately by two authors (Ke Du and Xi Zheng) and the following details were extracted: first author, publication date (year), country, sample size, sex and age of participants, sample source, and measurement method. The mean values of Mg concentration and standard deviation (SD) were recorded; otherwise, they were estimated from sample characteristics (size, median, and range) (Hozo et al., 2005). If there was a disagreement when extracting the data, it need to be discussed by all authors, and the final reasonable data was determined by the corresponding author. The nine-star Newcastle-Ottawa Scale (NOS) was used for quality assessment.

### Statistical Analysis

Statistical analyses were performed using STATA 12.0 (Stata, College Station, TX, USA). As the heterogeneity was significant, the results from the studies were combined using a random-effects model. The standardized mean difference (SMD), which standardizes the outcome for multiple studies to the effect size found in terms of the SD, was used as the summary statistic.

The Chi-square and I-square tests were used to assess heterogeneity. A subgroup analysis was then used to assess possible sources of heterogeneity, estimating the influences of different methods of Mg determination and different geographical locations of the populations. Meta-regression was also used to evaluate the moderating effect of variables on the meta-analysis outcome, including two study level characteristics (age and sex distribution) while the impact of the individual studies on the pooled SMD was assessed using sensitivity analysis. Potential publication bias was evaluated with Egger's and Begg's tests, as well as the “trim and fill” method. Sensitivity analysis was conducted to explore whether a significant difference in one study could markedly influence the overall outcome; this was done by eliminating successive individual studies from the repeated analysis. Finally, temporal effects were determined by a cumulative meta-analysis.

## Results

### Selection of Studies

**Figure 1:**
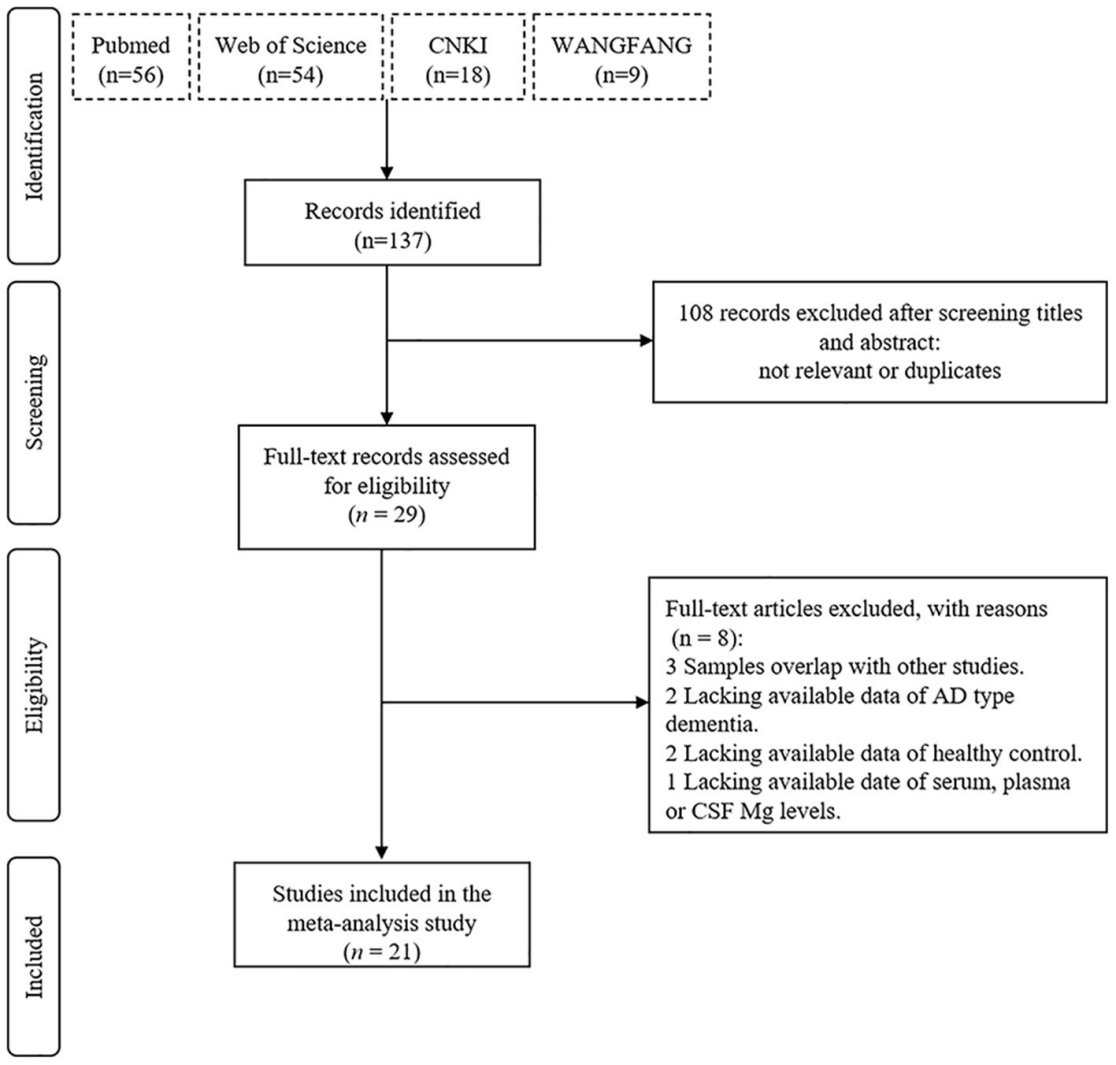
Flowchart of the selection process.

Twenty nine possible studies were totally found after a preliminary search in PubMed, Web of Science, CNKI, and WANGFANG. Eight articles were excluded for overlap in studies (*n* = 3), no AD type dementia (*n* = 2), no healthy control (*n* = 2), and unavailable serum, plasma, or CSF Mg levels (*n* = 1). As a result, 21 articles were included in the current analysis (1,112 AD cases and 1,001 HCs). Figure 1 presents a flowchart of the study selection.

In the included studies, the sample sizes varied between 8 and 174. The subjects were between 44.8 and 78.8 years old, with the proportion of female subjects between 0 and 75%. The geographical locations were Asia, Europe, and Africa. Mg concentrations were measured using atomic absorption spectrometry, inductively coupled plasma-atomic emission spectrometry, inductively coupled plasma-mass spectrometry, ion-selective electrode, and spectrophotometry. The average age was omitted in two studies. In addition, criteria for AD diagnosis were missing in two studies. The analytic method of Mg level in fluid were absent in one report. The details are listed in Table 1. The NOS quality assessment scale is shown in Supplementary Table 1. Most included studies were of high quality (18 high-quality and 3 moderate-quality studies).

**Table 1:** Characteristics of included studies in the meta-analysis.

			**AD Patients**					**Health Controls**				
**References**	**Year**	**Country**	***n***	**Gender**	**Age**	**Mg concentration**	**Criteria for AD Diagnosis**	***n***	**Gender**	**Age**	**Mg concentration**	**Method**
				**(% Female)**	**(Mean ± SD)**	**mean ± SD (mmol/L)**			**(% Female)**	**(Mean ± SD)**	**mean ± SD (mmol/L)**	
**Studies on serum**												
Zhu et al. (1997)	1997	China	8	0	75.0 ± 8.0	0.75 ± 0.04	DSM-IIIR	22	0	70.1 ± 7.4	0.83 ± 0.03	AAS
Cheng et al. (1999)	1999	China	53	52	78.8 ± 7.6	0.87 ± 0.07	DSM-IIIR	49	61	77.1 ± 4.3	0.85 ± 0.05	ICP-AES
Alimonti et al. (2007)	2007	Italy	53	68	74.5 ± 6.5	0.72 ± 0.03	NINCDS-ADRDA	124	35	44.8 ± 12.7	0.78 ± 0.02	ICP-AES
Cilliler et al. (2007)	2007	Turkey	37	54	-	0.92 ± 0.19	DSM-IV, NINCDS-ADRDA	34	-	-	1.00 ± 0.14	ICP-AES
Liu (2008)	2008	China	30	47	66.2 ± 9.9	0.041 ± 0.01	DSM-IV, NINCDS-ADRDA	28	46	66.8 ± 8.3	0.046 ± 0.01	ICP-AES
Gustaw-Rothenberg et al. (2010)	2010	Poland	30	-	69.1 ± 5.3	1.00 ± 0.24	DSM-IV, NINCDS-ADRDA	29	-	65.4 ± 3.7	0.782 ± 0.10	Spectrophotometry
Barbagallo et al. (2011)	2011	Italy	36	58	73.1 ± 0.9	0.50 ± 0.10	DSM-IV, NINCDS-ADRDA	65	59	73.8 ± 1.1	0.53 ± 0.10	ISE
Singh et al. (2014)	2014	India	100	47	62.7 ± 7.2	0.77 ± 0.12	NINCDS-ADRDA	100	39	59.7 ± 8.1	0.91 ± 0.21	Spectrophotometry
Wang (2015)	2015	China	57	33	71.2 ± 7.87	1.40 ± 0.36	DSM-IV, NINCDS-ADRDA	96	49	68.2 ± 7.7	1.45 ± 0.36	AAS
Zheng (2015)	2015	China	52	56	64.6 ± 8.96	1.00 ± 0.10	NINCDS-ADRDA	98	54	65.2 ± 7.2	0.98 ± 0.16	-
Koc et al. (2015)	2015	Turkey	44	49	77.7 ± 9.3	0.72 ± 0.17	DSM-IV, NINCDS-ADRDA	33	52	73.2 ± 10.6	0.67 ± 0.47	ICP-MS
Balmus et al. (2017)	2017	Romania	15	40	65.8 ± 3.9	0.39 ± 0.11	NINCDS-ADRDA	15	47	62.5 ± 3.4	0.54 ±0.09	AAS
studies on plasma												
Lemke (1995)	1995	Germany	12	67	77.5 ± 3.5	0.58 ± 0.07	DSM-IIIR	12	50	75.2 ± 6.4	0.7 ± 0.08	Spectrophotometry
Kurup and Kurup (2003)	2003	India	15	0	50–70	0.72 ± 0.05	-	15	0	50–70	0.99 ± 0.01	AAS
Bostrom et al. (2009b)	2009	Sweden	174	70	74 ± 5.7	0.89 ± 0.09	NINCDS-ADRDA	51	69	73 ± 6.8	0.88 ± 0.10	ICP-MS
Vural et al. (2010)	2010	Turkey	50	54	71.9 ± 6.8	0.784 ± 0.08	NINCDS-ADRDA	50	52	65.1 ± 7.1	0.876 ± 0.13	Spectrophotometry
Ahmed et al. (2017)	2017	Saudi Arabia	20	70	59.2 ± 8.3	0.38 ± 0.19	-	20	65	55.0 ± 5.2	1.02 ± 0.13	spectrophotometry
Xu et al. (2018)	2018	UK	42	48	78.2 ± 1.3	0.70 ± 0.06	NINCDS-ADRDA	43	46	78.1 ± 1.1	0.70 ± 0.07	ICP-MS
studies on CSF												
Bostrom et al. (2009a)	2009	Sweden	159	75	75.4 ± 6.8	1.15 ± 0.08	NINCDS-ADRDA	49	69	73.1 ± 7.7	1.18 ± 0.09	ICP-MS
Hozumi et al. (2011)	2011	Japan	21	38	-	1.32 ± 0.17	DSM-IV	15	60	-	1.23 ± 0.27	ICP-MS
Jouini et al. (2021)	2021	Tunisia	104	49	70.5 ± 7.5	1.13 ± 0.11	DSM-IV, NINCDS-ADRDA	53	53	68.5 ± 7.5	1.15 ± 0.05	Spectrophotometry

*NINCDS-ADRDA, National Institute of Neurological and Communicative Disorders and Stroke-Alzheimer's Disease and Related Disorders Association; DSM-IIIR or DSMIV, the Diagnostic and Statistical Manual for Mental Disorders; ICP-MS, inductively coupled plasma-mass spectrometry; ICP-AES, inductively coupled plasma-atomic emission spectrometry; AAS, atomic absorption spectrometry; ISE, ion-selective electrode*.

### Meta-Analysis of Mg Concentrations in Peripheral Blood

**Figure 2:**
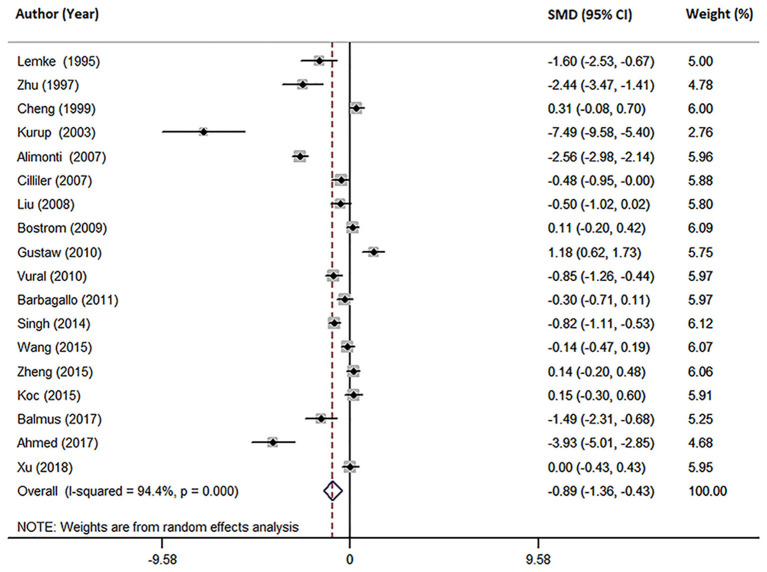
Forest plot of the random-effects meta-analysis of difference in peripheral blood Mg concentrations in AD patients and HCs. SMD, standardized mean difference; CI, confidence interval.

**Figure 3:**
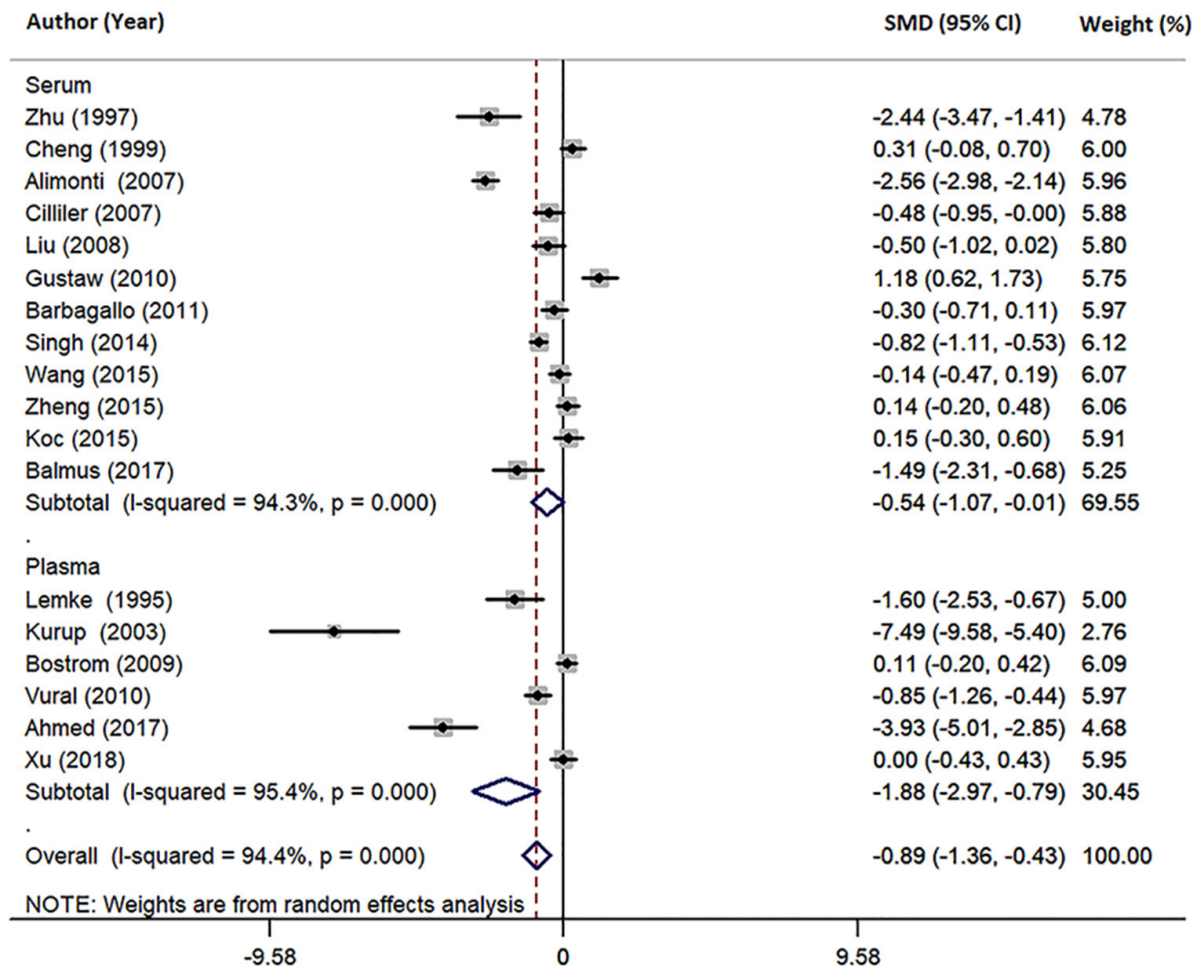
Forest plot of subgroup analysis by sample source on differences in serum and plasma Mg between AD and HCs. SMD, standardized mean difference; CI, confidence interval.

Eighteen studies measured peripheral blood Mg levels in both AD patients and HCs. The pooled sample size contained 828 AD patients and 884 HCs (Table 1). The results indicated that AD patients had less Mg levels in peripheral blood than HCs (SMD = −0.89; 95% CI [−1.36, −0.43]; *P* = 0.000; Figure 2). Heterogeneity among the included studies was observed (*I*^2^ = 94.4%, *P* = 0.000). According to sample source, subgroups analysis demonstrated significant heterogeneity in each subgroup (Table 2) and Mg levels were less in AD patients compared with HCs, in both serum subgroups (SMD = −0.54; 95% CI [−1.07, −0.01]; *P* = 0.045) and plasma subgroups (SMD = −1.88; 95% CI [−2.97, −0.79]; *P* = 0.001) (Figure 3). Additionally, according to the method of Mg measurement or geographical location, high heterogeneity still was found in subgroup analyses (Table 2). These results suggested that the sample source, Mg measurement method, and geographical location did not contribute to heterogeneity. Meta-regression analyses showed that neither the mean age nor the sex of AD patients affected Mg levels in peripheral blood (mean age: *P* = 0.282; sex: *P* = 0.103), while sensitivity analyses revealed that the individual study had no influence on the overall results (Supplementary Table 2). The cumulative analysis excluded the temporal effects affecting the results of the pooled analysis. Besides, Egger's (*P* = 0.031) and Begg's (*P* = 0.028) tests suggested that publication bias might be possible. Accordingly, the “trim and fill” method was used for sensitivity analysis, and it was observed that the general result was not significantly altered (SMD = −1.05; 95% CI [−1.55, −0.55]; *P* = 0.000), indicating a lack of impact by unpublished negative data.

**Table 2:** The subgroup analysis of studies reporting Mg levels in peripheral blood.

**Subgroups**	***n* of studies**	**SMD (95% CI)**	**I^**2**^**	***P*-value**
All studies	18	−0.89 (−1.36, −0.43)	94.4%	0.000
Methods
AAS	4	−2.62 (−4.59, −0.66)	95.5%	0.000
ICP-AES	4	−0.81 (−2.11, 0.49)	97.1%	0.000
Spectrophotometry	5	−1.12 (−2.19, −0.04)	95.2%	0.000
ICP-MS	3	0.09 (−0.13, 0.31)	0.0%	0.881
ISE	1	−0.30 (−0.71, −0.11)	-	-
-	1	0.14 (−0.20, 0.48)	-	-
Geographic locations
Asia	11	−1.01 (−1.54, −0.47)	93.1%	0.000
Europe	7	−0.65 (−1.58, 0.28)	96.2%	0.000

*ICP-MS, inductively coupled plasma-mass spectrometry; ICP-AES, inductively coupled plasma-atomic emission spectrometry; AAS, atomic absorption spectrometry; ISE, ion-selective electrode*.

### Meta-Analysis of Mg Levels in CSF Between AD and HCs

**Figure 4:**
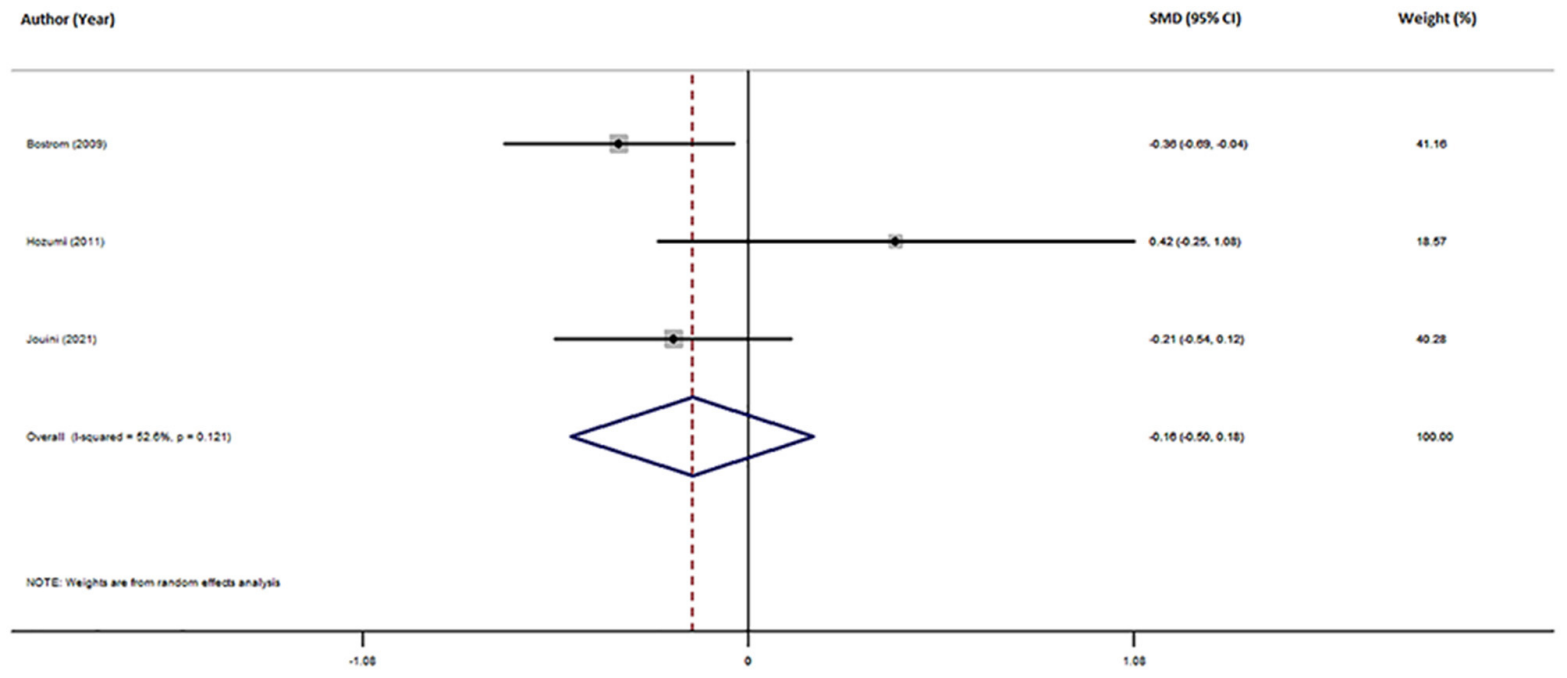
Forest plot of random-effects meta-analysis for differences in CSF Mg between AD and HCs. SMD, standardized mean difference; CI, confidence interval.

Three studies reported different CSF Mg levels in AD patients and HCs (Table 1). The pooled sample included 401 subjects, including 284 AD patients and 117 HCs. Patients with AD showed a tendency toward lower CSF Mg levels compared with HCs, although this difference was non-significant (SMD = −0.16; 95% CI [−0.50, 0.18]; *P* = 0.364; Figure 4). Due to the small number of studies, further analysis was not conducted.

### Meta-Analysis of Circulating Mg Levels in AD and HCs

**Figure 5:**
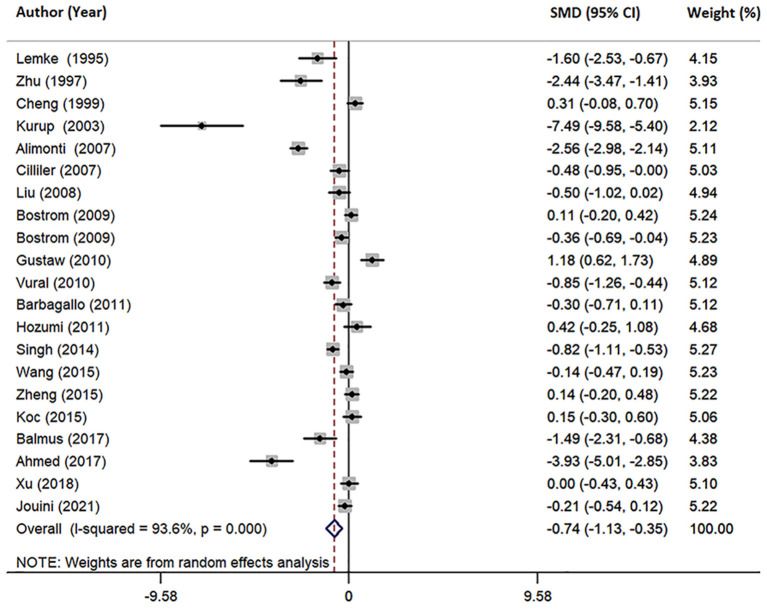
Forest plot of random-effects meta-analysis of differences in circulating Mg between AD and HCs. SMD, standardized mean difference; CI, confidence interval.

Furthermore, we conducted a joint analysis of 21 studies investigating circulating Mg levels, including serum, plasma and CSF. The pooled sample size contained 2,113 subjects, including 1,112 AD patients and 1,001 HCs (Table 1). The results revealed that circulating Mg levels in AD patients were significantly decreased compared with that in HCs (SMD = −0.74; 95% CI [−1.13, −0.35]; *P* = 0.000; Figure 5), in addition to high heterogeneity among these studies (*I*^2^ = 93.6%, *P*=0.006). Analysis of subgroups according to Mg measurement method and geographical location also showed significant heterogeneity (Table 3), suggesting that neither Mg measurement methods nor geographical location were the primary sources of heterogeneity. Neither the mean age nor sex of AD patients had obvious effects on circulating Mg levels by meta-regression analysis (mean age: *P* = 0.251; sex: *P* = 0.111), nor did individual studies influence the pooled SMD by sensitivity analysis (Supplementary Table 3). The cumulative meta-analysis did not show any significant temporal biases. Egger's (*P* = 0.046) and Begg's (*P* = 0.037) tests indicated the potential publication bias. However, sensitivity analysis using the “trim and fill” method showed that the conclusion was not substantially altered (SMD = −0.933; 95% CI [−1.35, −0.52]; *P* = 0.000), indicating that the results were statistically robust.

**Table 3:** The subgroup analysis of studies reporting circulating Mg levels.

**Subgroups**	***n* of studies**	**SMD (95% CI)**	**I^**2**^**	***P*-value**
All studies	21	−0.74 (−1.13, −0.35)	93.6%	0.000
Methods
AAS	4	−2.62 (−4.59, −0.66)	95.5%	0.000
ICP-AES	4	−0.81 (−2.11, 0.49)	97.1%	0.000
Spectrophotometry	6	−0.93 (−1.74, −0.11)	94.4%	0.000
ICP-MS	5	0.01 (−0.24, 0.25)	44.4%	0.126
ISE	1	−0.30 (−0.71, −0.11)	-	-
-	1	0.14 (−0.20, 0.48)	-	-
Geographic locations
Asia	12	−0.88 (−1.39, −0.37)	92.6%	0.000
Europe	8	−0.61 (−1.36, 0.15)	95.6%	0.000
Africa	1	−0.21 (−0.54, 0.12)	-	-

*ICP-MS, inductively coupled plasma-mass spectrometry; ICP-AES, inductively coupled plasma-atomic emission spectrometry; AAS, atomic absorption spectrometry; ISE, ion-selective electrode*.

## Discussion

The challenges associated with various nutritional deficiencies and the role of nutritional supplementation have received more attentions owing to the high incidence of AD in aging population throughout the world (Chiu et al., 2014; Shlisky et al., 2017; Tan et al., 2019). Mg is an essential mineral involved in many AD-associated biological processes (Toffa et al., 2019). However, previous reports about the circulating Mg status in AD still remain controversial. The major findings of this study revealed that Mg concentrations in peripheral blood were significantly lower in AD patients. Furthermore, it was also consistent with the joint meta-analysis performed on serum, plasma, and CSF levels, increasing the statistical power of our meta-analysis. In addition, although AD patients showed a tendency toward decreasing Mg concentrations in the CSF compared with HCs, the difference was not statistically significant. Because the sample size were limited for the studies on CSF Mg levels (three studies, including 284 AD patients and 117 HCs), further studies with large sample sizes are necessary to evaluate the CSF Mg levels in AD.

Consistent with our findings that AD patients present reduced circulating Mg levels, previous studies have suggested that Mg concentrations in the hair of AD patients were considerably less than controls (Kobayashi et al., 1989; Veronese et al., 2016). Additionally, Mg levels were lower in AD-affected brain areas such as Ammon's horn, entorhinal cortex, and frontal cortex (Andrasi et al., 2000, 2005). However, the mechanisms underlying the reduced brain levels of Mg remain unclear. The barrier function of the blood-brain barrier (BBB) is possibly damaged during aging and AD (Yamazaki and Kanekiyo, 2017). Therefore, the reduced Mg levels in AD-affected brains may be primarily attributed to disrupted BBB permeability. Nevertheless, the possibility of dietary deficiency cannot be ruled out, which requires further research to testify.

**Figure 6:**
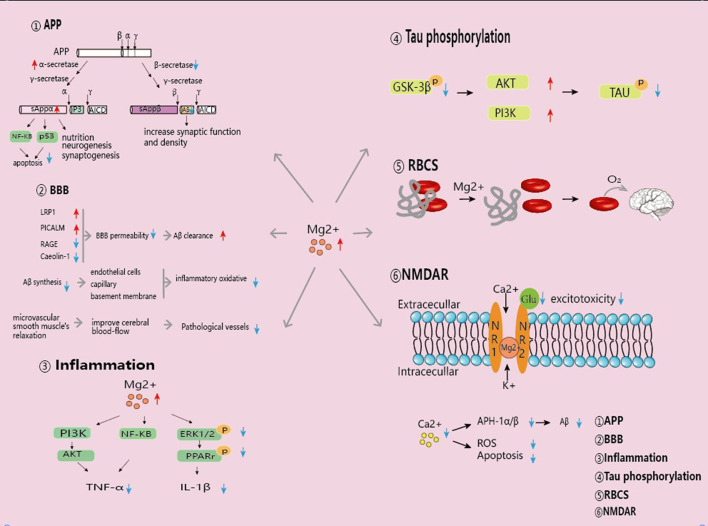
Mg is involved in multiple steps of AD pathogenesis.

The normal range of circulating Mg levels may be also influenced by age, sex, sample type, and geographical location. Therefore, we have reviewed those factors in each study included in this meta-analysis, and found that in most studies these factors were matched between the AD and control groups. Therefore, these factors should not influence our results. As studies included in this meta-analysis focused on the AD patients, it is difficult to confirm the causal association between Mg deficiency and AD. The dietary intake of AD patients is often poor in comparison with that of age-matched controls with normal cognitive function (Shatenstein et al., 2007). Therefore, Mg decrease may be a consequence of AD, possibly induced by malnutrition and poor nutrient intake. In addition to Mg, iron (Fe) and copper (Cu) are required for human health. Wang et al. did not observed the altered serum Fe levels in AD patients (Wang et al., 2015), whereas Ventriglia et al. described higher serum Cu concentrations in AD patients (Ventriglia et al., 2012). Therefore, these differences in circulating minerals could not be explained solely based on differences in dietary intake of AD patients. However, several studies have supported this causal relationship. For example, Cherbuin et al. reported that higher dietary Mg intake was associated with a lower risk of mild cognitive impairment (MCI) (Cherbuin et al., 2014). Glick and McMillan found that the cognitive decline associated with AD might be improved by increasing Mg dietary intake (Glick and McMillan, 2016). In addition, animal experiments have revealed the benefits of Mg supplementation on the performance of learning and memory. A study on rats pointed out that a formulated Mg compound administration increased the brain Mg levels and enhanced learning and working memory, as well as short-term synaptic facilitation and long-term potentiation (Slutsky et al., 2010). Increased Mg levels in the brain and plasma of elderly rats seemed to improve the maze performance and potentiation in the hippocampus after diet intake (Landfield and Morgan, 1984). Additionally, Yu et al. reported that high Mg concentration modulated Aβ protein precursor processing and reduced Aβ secretion in a mouse neuroblastoma cells (Yu et al., 2010). Mg is postulated to target multiple steps and various stages of AD pathogenesis (Figure 6). It has been shown to promote Aβ protein precursor α-cleavage (Yu et al., 2010), increase Aβ fibril clearance by regulating BBB permeability (Zhu et al., 2018), decrease tau hyperphosphorylation (Xu et al., 2014), inhibit Aβ-induced neuroinflammation (Wang et al., 2017; Yu et al., 2018), disrupt RBC (red blood cell)-fibrin aggregates which promotes oxygen delivery to the brain (Lipinski and Pretorius, 2013), and prevent the downregulation of N-methyl-D-aspartate receptors in AD models (Li et al., 2014; Huang et al., 2018) (Table 4). The present study increased statistical power by combining the results of different studies, and showed that AD patients have a poor circulating Mg status, further supporting the hypothesis that Mg deficiency is an AD risk factor. Based on these findings, clinical trials are demanded to explore the potential effects of Mg for AD prevention or treatment.

**Table 4:** Mg intervention against pathological phenomena involved in AD.

**Reagent**	***In vivo* model**	***In vitro* model**	**Mechanism**	**Intervention effect**	**References**
Magnesium-L-threonate	Aged rats	-	Enhance NMDAR-dependent signaling	Enhance both short-term synaptic facilitation and long-term potentiation and improve learning and memory functions	Slutsky et al., 2010
Magnesium sulfate	-	N2a cells stably expressing PS1 and APP	Promote Aβ protein precursor α-cleavage and modulate APP retention on cell surface	Reduce Aβ production and demote the amyloidogenic processing	Yu et al., 2010
Magnesium sulfate and magnesium chloride	-	BBB model constructed from endothelial cells and astrocytes	Suppress Caveolin-1 and RAGE while promote PICALM and LRP1 expression	Reduce BBB permeability and regulates Aβ transcytosis	Zhu et al., 2018
Magnesium sulfate	Streptozotocin-induced sporadic AD rats	-	Inhibit GSK-3β, increase the activity of AKT and PI3K	Decrease tau hyperphosphorylation, and protect cognitive function and synaptic plasticity	Xu et al., 2014
Magnesium-L-threonate	APP/PS1 mice	Aβ or IL-1β -induced Glioblastoma A172 and mouse brain D1A glial cells	Activate ERK1/2 and PPARγ signaling pathways	Reduce IL-1β expression and neuroinflammation	Wang et al., 2017
Magnesium-L-threonate	APP/PS1 mice	Human- or mouse-derived glial and neuronal cell lines	Activate PI3K/AKT signaling pathway and inhibit NF-kB signaling pathway	Inhibit TNF-α expression and reduce the toxic β-fragmentation of APP	Yu et al., 2018
Magnesium chloride	-	Whole blood smears of AD patients with or without added Mg	Disrupt RBC-parafibrin aggregate	Allow erythrocytes to return to the circulation and promote oxygen delivery to the brain	Lipinski and Pretorius, 2013
Magnesium-L-threonate	APP/PS1 mice	-	Protect NMDAR signaling and reduce the expression of BACE1	Prevent/reverse learning and memory deterioration	Li et al., 2014
Magnesium-L-threonate	APP/PS1 mice	-	Activate CaMKII and CREB activation	Improve recognition and spatial memory	Huang et al., 2018

*NMDAR, N-methyl-D-aspartate receptor; PS1, presenilin 1; APP, amyloid-β protein precursor; BBB, blood-brain barrier; RAGE, receptor for advanced glycation end products; PICALM, phosphatidylinositol binding clathrin assembly protein; LRP1, low-density lipoprotein receptor-related protein 1; GSK-3β, glycogen synthase kinase-3β; ERK, extracellular signal-regulated protein kinases; PPAR, peroxisome proliferator-activated receptor; NF-kB, nuclear factor-kB; TNF-α, tumor necrosis factor-α; RBC, red blood cells; BACE1, β-site amyloid precursor protein-cleaving enzyme 1; CaMKII, calcium-calmodulin dependent protein kinase II; CREB, cAMP-response element binding protein*.

This meta-analysis has a few limitations. First, there are still few reports on CSF Mg levels although we have performed possibly comprehensive searches. Additional investigations with larger samples are required to confirm our findings. Second, circulating Mg levels varied among included studies. Typically, variations occur due to different techniques used for sampling or the analytic methods. Third, we just searched studies written in English or Chinese, studies in other languages were not included. Fourth, the high degree of heterogeneity among the studies necessitates cautious interpretation.

## Conclusions

In summary, our analysis concluded that circulating Mg levels in AD patients were significantly lower than those in HCs, providing more evidence that Mg supplementation or Mg rich diets possibly exerted a promising preventive or therapeutic strategies for treating AD patients with a poorer Mg status.

## Data Availability Statement

The original contributions presented in the study are included in the article/Supplementary Material, further inquiries can be directed to the corresponding author/s.

## Author Contributions

KD and M-YL contributed to the conception and design of the study. KD and XZ searched the databases, analyzed the data, and drafted the manuscript. KD, XZ, Z-TM, J-YL, and W-JJ screened the publications, conducted the quality assessment of the included studies, and extracted the data. M-YL had primary responsibility for the final content. All authors contributed to the writing, reviewing, and revising of the manuscript and read and approved the final manuscript.

## Funding

This study was supported by grants from National Natural Science Foundation of China (No. 81603112), Natural Science Foundation of Liaoning Province (2020-MS-161), Key Project of Basic Scientific Research project of Higher Education in Liaoning Province (LJKZ0775), and Liaoning Province Key Research and Development Plan guiding project (2018225089).

## Conflict of Interest

The authors declare that the research was conducted in the absence of any commercial or financial relationships that could be construed as a potential conflict of interest.

## Publisher's Note

All claims expressed in this article are solely those of the authors and do not necessarily represent those of their affiliated organizations, or those of the publisher, the editors and the reviewers. Any product that may be evaluated in this article, or claim that may be made by its manufacturer, is not guaranteed or endorsed by the publisher.

## Abbreviations

AD: Alzheimer's disease
AAS: atomic absorption spectrometry
CI: confidence interval
CNKI: China National Knowledge Infrastructure
CSF: cerebrospinal fluid
HCs: healthy controls
ICP-AES: inductively coupled plasma-atomic emission spectrometry
ICP-MS: inductively coupled plasma-mass spectrometry
ISE: ion-selective electrode
SMD: standardized mean difference.

## References

[B1] Abdullahi I. Watila M. M. Shahi N. Nyandaiti Y. W. Bwala S. A. (2019). Serum magnesium in adult patients with idiopathic and symptomatic epilepsy in Maiduguri, Northeast Nigeria. Niger J. Clin. Pract. 22, 186–193. 10.4103/njcp.njcp_252_18 30729941

[B2] Ahmed A. S. Elgharabawy R. M. Al-Najjar A. H. (2017). Ameliorating effect of anti-Alzheimer's drugs on the bidirectional association between type 2 diabetes mellitus and Alzheimer's disease. Exp. Biol. Med. (Maywood) 242, 1335–1344. 10.1177/1535370217711440 28534431 PMC5529002

[B3] Akiyama H. Barger S. Barnum S. Bradt B. Bauer J. Cole G. M. (2000). Inflammation and Alzheimer's disease. Neurobiol. Aging 21, 383–421. 10.1016/S0197-4580(00)00124-X 10858586 PMC3887148

[B4] Alimonti A. Ristori G. Giubilei F. Stazi M. A. Pino A. Visconti A. . (2007). Serum chemical elements and oxidative status in Alzheimer's disease, Parkinson disease and multiple sclerosis. Neurotoxicology 28, 450–456. 10.1016/j.neuro.2006.12.001 17267042

[B5] Andrasi E. Igaz S. Molnar Z. Mako S. (2000). Disturbances of magnesium concentrations in various brain areas in Alzheimer's disease. Magnes. Res. 13, 189–196. 11008926

[B6] Andrasi E. Pali N. Molnar Z. Kosel S. (2005). Brain aluminum, magnesium and phosphorus contents of control and Alzheimer-diseased patients. J. Alzheimers Dis. 7, 273–284. 10.3233/JAD-2005-7402 16131728

[B7] Balmus I. M. Strungaru S. A. Ciobica A. Nicoara M. N. Dobrin R. Plavan G. . (2017). Preliminary data on the interaction between some biometals and oxidative stress status in mild cognitive impairment and Alzheimer's disease patients. Oxid. Med. Cell Longev. 2017:7156928. 10.1155/2017/7156928 28811866 PMC5546061

[B8] Barbagallo M. Belvedere M. Di Bella G. Dominguez L. J. (2011). Altered ionized magnesium levels in mild-to-moderate Alzheimer's disease. Magnes. Res. 24, S115–S121. 10.1684/mrh.2011.0287 21951617

[B9] Belaidi A. A. Bush A. I. (2016). Iron neurochemistry in Alzheimer's disease and Parkinson's disease: targets for therapeutics. J. Neurochem. 139, 179–197. 10.1111/jnc.13425 26545340

[B10] Bostrom F. Hansson O. Blennow K. Gerhardsson L. Lundh T. Minthon L. . (2009a). Cerebrospinal fluid total tau is associated with shorter survival in dementia with Lewy bodies. Dement. Geriatr. Cogn. Disord. 28, 314–319. 10.1159/000249145 19844105

[B11] Bostrom F. Hansson O. Gerhardsson L. Lundh T. Minthon L. Stomrud E. . (2009b). CSF Mg and Ca as diagnostic markers for dementia with Lewy bodies. Neurobiol. Aging 30, 1265–1271. 10.1016/j.neurobiolaging.2007.10.018 18191875

[B12] Cheng Y. M. Ge W. Zhang S. Y. Zhu J. Z. He T. M. Ye S. L. (1999). Study on calcium, magnesium, zinc and aluminum content in serum of senile dementia patients. Bull. Sci. Technol. 15, 467–469.

[B13] Cherbuin N. Kumar R. Sachdev P. S. Anstey K. J. (2014). Dietary mineral intake and risk of mild cognitive impairment: the PATH through life project. Front. Aging Neurosci. 6:4. 10.3389/fnagi.2014.00004 24550825 PMC3912433

[B14] Chiu H. Y. Yeh T. H. Huang Y. C. Chen P. Y. (2016). Effects of intravenous and oral magnesium on reducing migraine: a meta-analysis of randomized controlled trials. Pain Phys. 19, E97–E112. 10.36076/ppj/2016.19.E97 26752497

[B15] Chiu S. Woodbury-Farina M. A. Shad M. U. Husni M. Copen J. Bureau Y. . (2014). The role of nutrient-based epigenetic changes in buffering against stress, aging, and Alzheimer's disease. Psychiatr. Clin. North Am. 37, 591–623. 10.1016/j.psc.2014.09.001 25455068

[B16] Cilliler A. E. Ozturk S. Ozbakir S. (2007). Serum magnesium level and clinical deterioration in Alzheimer's disease. Gerontology 53, 419–422. 10.1159/000110873 17992016

[B17] Dolati S. Rikhtegar R. Mehdizadeh A. Yousefi M. (2020). The role of magnesium in pathophysiology and migraine treatment. Biol. Trace Elem. Res. 196, 375–383. 10.1007/s12011-019-01931-z 31691193

[B18] Donnelly P. S. Xiao Z. Wedd A. G. (2007). Copper and Alzheimer's disease. Curr. Opin. Chem. Biol. 11, 128–133. 10.1016/j.cbpa.2007.01.678 17300982

[B19] Du K. Liu M. Pan Y. Zhong X. Wei M. (2017). Association of serum manganese levels with Alzheimer's disease and mild cognitive impairment: a systematic review and meta-analysis. Nutrients 9:231. 10.3390/nu9030231 28273828 PMC5372894

[B20] Glick J. L. McMillan P. A. (2016). A multipronged, nutritional-based strategy for managing Alzheimer's disease. Med. Hypotheses. 91, 98–102. 10.1016/j.mehy.2016.04.007 27142155

[B21] Gonzalez-Dominguez R. Garcia-Barrera T. Gomez-Ariza J. L. (2014). Characterization of metal profiles in serum during the progression of Alzheimer's disease. Metallomics 6, 292–300. 10.1039/C3MT00301A 24343096

[B22] Gustaw-Rothenberg K. Kowalczuk K. Stryjecka-Zimmer M. (2010). Lipids' peroxidation markers in Alzheimer's disease and vascular dementia. Geriatr. Gerontol. Int. 10, 161–166. 10.1111/j.1447-0594.2009.00571.x 20446930

[B23] Hozo S. P. Djulbegovic B. Hozo I. (2005). Estimating the mean and variance from the median, range, and the size of a sample. BMC Med. Res. Methodol. 5:13. 10.1186/1471-2288-5-13 15840177 PMC1097734

[B24] Hozumi I. Hasegawa T. Honda A. Ozawa K. Hayashi Y. Hashimoto K. . (2011). Patterns of levels of biological metals in CSF differ among neurodegenerative diseases. J. Neurol. Sci. 303, 95–99. 10.1016/j.jns.2011.01.003 21292280

[B25] Huang Y. Huang X. Zhang L. Han F. Pang K. L. Li X. . (2018). Magnesium boosts the memory restorative effect of environmental enrichment in Alzheimer's disease mice. CNS Neurosci. Ther. 24, 70–79. 10.1111/cns.12775 29125684 PMC6489792

[B26] Jouini N. Saied Z. Ben Sassi S. Nebli F. Messaoud T. Hentati F. . (2021). Impacts of iron metabolism dysregulation on Alzheimer's disease. J. Alzheimers Dis. 80, 1439–1450. 10.3233/JAD-201250 33682709

[B27] Kawahara M. Tanaka K. I. Kato-Negishi M. (2018). Zinc, carnosine, and neurodegenerative diseases. Nutrients 10:147. 10.3390/nu10020147 29382141 PMC5852723

[B28] Kirkland A. E. Sarlo G. L. Holton K. F. (2018). The role of magnesium in neurological disorders. Nutrients 10:730. 10.3390/nu10060730 29882776 PMC6024559

[B29] Kobayashi S. Fujiwara S. Arimoto S. Koide H. Fukuda J. Shimode K. . (1989). Hair aluminium in normal aged and senile dementia of Alzheimer type. Prog. Clin. Biol. Res. 317, 1095–1109. 2602406

[B30] Koc E. R. Ilhan A. Zubeyde A. Acar B. Gurler M. Altuntas A. . (2015). A comparison of hair and serum trace elements in patients with Alzheimer disease and healthy participants. Turk. J. Med. Sci. 45, 1034–1039. 10.3906/sag-1407-67 26738344

[B31] Kurup R. K. Kurup P. A. (2003). Hypothalamic digoxin, hemispheric chemical dominance, and Alzheimer's disease. Int. J. Neurosci. 113, 361–381. 10.1080/00207450390162146 12803139

[B32] Landfield P. W. Morgan G. A. (1984). Chronically elevating plasma Mg2+ improves hippocampal frequency potentiation and reversal learning in aged and young rats. Brain Res. 322, 167–171. 10.1016/0006-8993(84)91199-5 6097334

[B33] Lane D. J. R. Ayton S. Bush A. I. (2018). Iron and Alzheimer's disease: an update on emerging mechanisms. J. Alzheimers Dis. 64, S379–S395. 10.3233/JAD-179944 29865061

[B34] Lemke M. R. (1995). Plasma magnesium decrease and altered calcium/magnesium ratio in severe dementia of the Alzheimer type. Biol. Psychiatry 37, 341–343. 10.1016/0006-3223(94)00241-T 7748988

[B35] Lesne S. Koh M. T. Kotilinek L. Kayed R. Glabe C. G. Yang A. . (2006). A specific amyloid-beta protein assembly in the brain impairs memory. Nature 440, 352–357. 10.1038/nature04533 16541076

[B36] Li W. Yu J. Liu Y. Huang X. Abumaria N. Zhu Y. . (2014). Elevation of brain magnesium prevents synaptic loss and reverses cognitive deficits in Alzheimer's disease mouse model. Mol. Brain 7:65. 10.1186/s13041-014-0065-y 25213836 PMC4172865

[B37] Lipinski B. Pretorius E. (2013). The role of iron-induced fibrin in the pathogenesis of Alzheimer's disease and the protective role of magnesium. Front. Hum. Neurosci. 7:735. 10.3389/fnhum.2013.00735 24194714 PMC3810650

[B38] Liu K. B. (2008). The Study of the Association Between Trace Element and Senile Dementia/Depressive Disorder. Jinan: Shandong University.

[B39] Mezzaroba L. Alfieri D. F. Colado Simao A. N. Vissoci Reiche E. M. (2019). The role of zinc, copper, manganese and iron in neurodegenerative diseases. Neurotoxicology 74 230–241. 10.1016/j.neuro.2019.07.007 31377220

[B40] Moher D. Liberati A. Tetzlaff J. Altman D. G. (2010). Preferred reporting items for systematic reviews and meta-analyses: the PRISMA statement. Int. J. Surg. 8, 336–341. 10.1016/j.ijsu.2010.02.007 20171303

[B41] Oyanagi K. Hashimoto T. (2011). “Magnesium in Parkinson's disease: an update in clinical and basic aspects,” in Magnesium in the Central Nervous System, eds R. Vink and M. Nechifor (Adelaide: Cambridge University Press). p. 229–236. 29920021

[B42] Sensi S. L. Granzotto A. Siotto M. Squitti R. (2018). Copper and zinc dysregulation in Alzheimer's Disease. Trends Pharmacol. Sci. 39, 1049–1063. 10.1016/j.tips.2018.10.001 30352697

[B43] Sharma K. (2019). Cholinesterase inhibitors as Alzheimer's therapeutics (Review). Mol. Med. Rep. 20, 1479–1487. 10.3892/mmr.2019.10374 31257471 PMC6625431

[B44] Shatenstein B. Kergoat M. J. Reid I. (2007). Poor nutrient intakes during 1-year follow-up with community-dwelling older adults with early-stage Alzheimer dementia compared to cognitively intact matched controls. J. Am. Diet Assoc. 107, 2091–2099. 10.1016/j.jada.2007.09.008 18060894

[B45] Shen Y. Dai L. Tian H. Xu R. Li F. Li Z. . (2019). Treatment of magnesium-L-threonate elevates the magnesium level in the cerebrospinal fluid and attenuates motor deficits and dopamine neuron loss in a mouse model Of Parkinson's disease. Neuropsychiatr. Dis. Treat. 15, 3143–3153. 10.2147/NDT.S230688 31806980 PMC6857673

[B46] Shlisky J. Bloom D. E. Beaudreault A. R. Tucker K. L. Keller H. H. Freund-Levi Y. . (2017). Nutritional considerations for healthy aging and reduction in age-related chronic disease. Adv. Nutr. 8, 17–26. 10.3945/an.116.013474 28096124 PMC5227979

[B47] Singh N. K. Banerjee B. D. Bala K. Basu M. Chhillar N. (2014). Polymorphism in cytochrome P450 2D6, glutathione S-transferases Pi 1 genes, and organochlorine pesticides in alzheimer disease: a case-control study in North Indian population. J. Geriatr. Psychiatry Neurol. 27, 119–127. 10.1177/0891988714522698 24584466

[B48] Slutsky I. Abumaria N. Wu L. J. Huang C. Zhang L. Li B. . (2010). Enhancement of learning and memory by elevating brain magnesium. Neuron 65, 165–177. 10.1016/j.neuron.2009.12.026 20152124

[B49] Tan X. Zhang Y. Shao H. (2019). Healthy China 2030, a breakthrough for improving health. Glob. Health Promot. 26, 96–99. 10.1177/1757975917743533 29297762

[B50] Toffa D. H. Magnerou M. A. Kassab A. Hassane Djibo F. Sow A. D. (2019). Can magnesium reduce central neurodegeneration in Alzheimer's disease? Basic evidences and research needs. Neurochem. Int. 126, 195–202. 10.1016/j.neuint.2019.03.014 30905744

[B51] Ventriglia M. Brewer G. J. Simonelli I. Mariani S. Siotto M. Bucossi S. . (2015). Zinc in Alzheimer's disease: A meta-analysis of serum, plasma, and cerebrospinal fluid studies. J. Alzheimers Dis. 46, 75–87. 10.3233/JAD-141296 25697706

[B52] Ventriglia M. Bucossi S. Panetta V. Squitti R. (2012). Copper in Alzheimer's disease: a meta-analysis of serum, plasma, and cerebrospinal fluid studies. J. Alzheimers Dis. 30, 981–984. 10.3233/JAD-2012-120244 22475798

[B53] Veronese N. Zurlo A. Solmi M. Luchini C. Trevisan C. Bano G. . (2016). Magnesium status in Alzheimer's disease: a systematic review. Am. J. Alzheimers Dis. Other Dement. 31, 208–213. 10.1177/1533317515602674 26351088 PMC10852887

[B54] Vural H. Demirin H. Kara Y. Eren I. Delibas N. (2010). Alterations of plasma magnesium, copper, zinc, iron and selenium concentrations and some related erythrocyte antioxidant enzyme activities in patients with Alzheimer's disease. J. Trace Elem. Med. Biol. 24, 169–173. 10.1016/j.jtemb.2010.02.002 20569929

[B55] Wang L. Z. (2015). A Case-Control Study for Relevant Factors of Alzheimer's Disease. Huhhot: Inner Mongolia Medical University.

[B56] Wang P. Yu X. Guan P. P. Guo J. W. Wang Y. Zhang Y. . (2017). Magnesium ion influx reduces neuroinflammation in Abeta precursor protein/Presenilin 1 transgenic mice by suppressing the expression of interleukin-1beta. Cell Mol. Immunol. 14, 451–464. 10.1038/cmi.2015.93 26549801 PMC5423087

[B57] Wang Z. X. Tan L. Wang H. F. Ma J. Liu J. Tan M. S. . (2015). Serum iron, zinc, and copper levels in patients with alzheimer's disease: a replication study and meta-analyses. J. Alzheimers Dis. 47, 565–581. 10.3233/JAD-143108 26401693

[B58] Xu J. Church S. J. Patassini S. Begley P. Kellett K. A. B. Vardy E. . (2018). Plasma metals as potential biomarkers in dementia: a case-control study in patients with sporadic Alzheimer's disease. Biometals 31, 267–276. 10.1007/s10534-018-0089-3 29516299 PMC5978903

[B59] Xu Z. P. Li L. Bao J. Wang Z. H. Zeng J. Liu E. J. . (2014). Magnesium protects cognitive functions and synaptic plasticity in streptozotocin-induced sporadic Alzheimer's model. PLoS ONE 9:e108645. 10.1371/journal.pone.0108645 25268773 PMC4182554

[B60] Yamazaki Y. Kanekiyo T. (2017). Blood-brain barrier dysfunction and the pathogenesis of Alzheimer's disease. Int. J. Mol. Sci. 18:1965. 10.3390/ijms18091965 28902142 PMC5618614

[B61] Yary T. Kauhanen J. (2019). Dietary intake of magnesium and the risk of epilepsy in middle-aged and older Finnish men: a 22-year follow-up study in a general population. Nutrition 58, 36–39. 10.1016/j.nut.2018.06.019 30273823

[B62] Yu J. Sun M. Chen Z. Lu J. Liu Y. Zhou L. . (2010). Magnesium modulates amyloid-beta protein precursor trafficking and processing. J. Alzheimers Dis. 20, 1091–1106. 10.3233/JAD-2010-091444 20413885

[B63] Yu X. Guan P. P. Zhu D. Liang Y. Y. Wang T. Wang Z. Y. . (2018). Magnesium ions inhibit the expression of tumor necrosis factor alpha and the activity of gamma-secretase in a beta-amyloid protein-dependent mechanism in APP/PS1 transgenic mice. Front. Mol. Neurosci. 11:172. 10.3389/fnmol.2018.00172 29899688 PMC5988891

[B64] Zheng F. (2015). A case-control study on related influence factors of Alzheimer's disease. Changchun: Jilin University.

[B65] Zhu D. Su Y. Fu B. Xu H. (2018). Magnesium reduces blood-brain barrier permeability and regulates amyloid-beta transcytosis. Mol. Neurobiol. 55, 7118–7131. 10.1007/s12035-018-0896-0 29383689

[B66] Zhu M. W. Tang H. C. Zhao L. (1997). Determination of trace elements in serum of senile dementia. Stud. Trace Elem. Health 14, 18–19.

