# Supplementary material for: Association of Circulating Magnesium Levels in Patients With Alzheimer's Disease From 1991 to 2021: A Systematic Review and Meta-Analysis
Source: Front Aging Neurosci. 2022 Jan 10;13:799824. doi: 10.3389/fnagi.2021.799824 (PMC8784804; doi:10.3389/fnagi.2021.799824)
Supplement: Supplementary file 1 [file Table_1.DOCX]

**Association of Circulating Magnesium Levels in Patients with Alzheimer's Disease From 1991 to 2021: A Systematic Review and Meta‐Analysis**

Ke Du, Xi Zheng, Wen-Juan Jiang, et al.

**Supplementary Material**

**Contents**

**Supplemental Methods:** Search strategy

**Supplemental Table 1** Newcastle Ottawa Scale scores of the included studies

**Supplemental Table 2** Sensitivity analyses for Mg levels in peripheral blood comparing AD Versus HC: excluding one study at a time

**Supplemental Table 3** Sensitivity analyses for circulating Mg levels comparing AD Versus HC: excluding one study at a time

**Supplemental Reference**

**Supplemental Methods**

**Search strategy**

| **No.** | **Search Terms** |
| --- | --- |
| #1 | “Alzheimer’s disease (Title/Abstract) |
| #2 | “magnesium” (Title/Abstract) |
| #3 | “serum” (Title/Abstract) OR “plasma” (Title/Abstract) OR “ cerebrospinal fluid” (Title/Abstract) |
| #4 | Combination 1 AND 2 AND 3 |

**Supplemental Table 1** Newcastle Ottawa Scale scores of the included studies

| **ID** | **Author** | **Year** | **Country** | **Selection** | | | | **Comparability** | | **Exposure** | | | | **Overall Quality** | |
| --- | --- | --- | --- | --- | --- | --- | --- | --- | --- | --- | --- | --- | --- | --- | --- |
|  |  |  |  | 1 | 2 | 3 | 4 | 5A | 5B | 6 | 7 | 8 |  | |  |
| 1 | Zhu [1] | 1997 | China | * |  | * | * | * |  | * | * |  | 6 | |  |
| 2 | Cheng [2] | 1999 | China | * |  | * | * | * | * | * | * |  | 7 | |  |
| 3 | Alimonti [3] | 2007 | Italy | * | * | * | * | * | * | * | * |  | 8 | |  |
| 4 | Cilliler [4] | 2007 | Turkey | * | * | * | * |  | * | * | * |  | 7 | |  |
| 5 | Liu [5] | 2008 | China | * | * | * | * | * | * | * | * |  | 8 | |  |
| 6 | Gustaw [6] | 2010 | Poland | * |  | * | * | * | * | * | * |  | 7 | |  |
| 7 | Barbagallo [7] | 2011 | Italy | * | * | * | * | * | * | * | * |  | 8 | |  |
| 8 | Singh [8] | 2014 | India | * | * | * | * | * | * | * | * |  | 8 | |  |
| 9 | Wang [9] | 2015 | China | * | * | * | * | * |  | * | * |  | 7 | |  |
| 10 | Zheng [10] | 2015 | China | * | * | * | * | * | * | * |  |  | 7 | |  |
| 11 | Koc [11] | 2015 | Turkey | * |  | * | * | * | * | * | * |  | 7 | |  |
| 12 | Balmus [12] | 2017 | Romania | * |  | * | * | * |  | * | * |  | 6 | |  |
| 13 | Lemke [13] | 1995 | Germany | * | * | * | * | * | * | * | * |  | 8 | |  |
| 14 | Kurup [14] | 2003 | India | * |  | * | * | * | * | * | * |  | 7 | |  |
| 15 | Bostrom [15] | 2009 | Sweden | * | * | * | * | * |  | * | * |  | 7 | |  |
| 16 | Vural [16] | 2010 | Turkey | * |  | * | * | * | * | * | * |  | 7 | |  |
| 17 | Ahmed [17] | 2017 | SAU | * |  | * | * | * | * | * | * |  | 7 | |  |
| 18 | Xu [18] | 2018 | UK | * |  | * | * | * | * | * | * |  | 7 | |  |
| 19 | Bostrom [19] | 2009 | Sweden | * | * | * | * | * | * | * | * |  | 8 | |  |
| 20 | Hozumi [20] | 2011 | Japan | * |  | * | * |  | * | * | * |  | 6 | |  |
| 21 | Jouini [21] | 2021 | Tunisia | * |  | * | * | * | * | * | * |  | 7 | |  |

1. Case definition is sufficient, with independent verification. 2. Continuous collection and representative cases. 3. Community control. 4. Control of disease history without neurological system. 5 A. Research controls age. 5 B. Research controls other confounding factors. 6. Exposure is determined by reliable records. 7. The same method was used to determine the exposure of the case group and the control group. 8. None-response rates were similar for case and control groups. A study can be awarded a maximum of one star for each numbered item within the Selection and Exposure categories and a maximum of two stars for Comparability. Scores for low (0–3), moderate (4–6), and high-quality studies (7–9) were assigned.

**Supplemental Table 2** Sensitivity analyses for Mg levels in peripheral blood comparing AD Versus HC: excluding one study at a time^1^

| **Study omitted** |  | **Estimate** |  | **95% Confidence Interval** | | |
| --- | --- | --- | --- | --- | --- | --- |
| Zhu [1] |  | -0.81 |  | -1.28 |  | -0.34 |
| Cheng [2] |  | -0.97 |  | -1.46 |  | -0.49 |
| Alimonti [3] |  | -0.72 |  | -1.13 |  | -0.32 |
| Cilliler [4] |  | -0.93 |  | -1.42 |  | -0.44 |
| Liu [5] |  | -0.92 |  | -1.41 |  | -0.44 |
| Gustaw [6] |  | -1.01 |  | -1.47 |  | -0.55 |
| Barbagallo [7] |  | -0.94 |  | -1.44 |  | -0.45 |
| Singh [8] |  | -0.91 |  | -1.42 |  | -0.41 |
| Wang [9] |  | -0.96 |  | -1.46 |  | -0.45 |
| Zheng [10] |  | -0.97 |  | -1.46 |  | -0.48 |
| Koc [11] |  | -0.96 |  | -1.45 |  | -0.48 |
| Balmus [12] |  | -0.86 |  | -1.33 |  | -0.38 |
| Lemke [13] |  | -0.85 |  | -1.33 |  | -0.38 |
| Kurup [14] |  | -0.70 |  | -1.13 |  | -0.26 |
| Bostrom [15] |  | -0.97 |  | -1.47 |  | -0.47 |
| Vural [16] |  | -0.90 |  | -1.40 |  | -0.41 |
| Ahmed [17] |  | -0.73 |  | -1.17 |  | -0.28 |
| Xu [18] |  | -0.96 |  | -1.45 |  | -0.47 |

^1^Effect sizes were pooled using random-effects mode

**Supplemental Table 3** Sensitivity analyses for circulating Mg levels comparing AD Versus HC: excluding one study at a time^1^

| **Study omitted** |  | **Estimate** |  | **95% Confidence Interval** | | |
| --- | --- | --- | --- | --- | --- | --- |
| Zhu [1] |  | -0.67 |  | -1.06 |  | -0.28 |
| Cheng [2] |  | -0.80 |  | -1.21 |  | -0.40 |
| Alimonti [3] |  | -0.59 |  | -0.92 |  | -0.25 |
| Cilliler [4] |  | -0.76 |  | -1.17 |  | -0.35 |
| Liu [5] |  | -0.76 |  | -1.17 |  | -0.35 |
| Gustaw [6] |  | -0.83 |  | -1.22 |  | -0.45 |
| Barbagallo [7] |  | -0.77 |  | -1.19 |  | -0.36 |
| Singh [8] |  | -0.75 |  | -1.17 |  | -0.33 |
| Wang [9] |  | -0.79 |  | -1.20 |  | -0.37 |
| Zheng [10] |  | -0.80 |  | -1.21 |  | -0.39 |
| Koc [11] |  | -0.79 |  | -1.20 |  | -0.39 |
| Balmus [12] |  | -0.71 |  | -1.10 |  | -0.31 |
| Lemke [13] |  | -0.70 |  | -1.10 |  | -0.31 |
| Kurup [14] |  | -0.59 |  | -0.95 |  | -0.22 |
| Bostrom [15] |  | -0.80 |  | -1.21 |  | -0.39 |
| Vural [16] |  | -0.74 |  | -1.15 |  | -0.33 |
| Ahmed [17] |  | -0.60 |  | -0.97 |  | -0.23 |
| Xu [18] |  | -0.79 |  | -1.20 |  | -0.38 |
| Bostrom [19] |  | -0.78 |  | -1.19 |  | -0.36 |
| Hozumi [20] |  | -0.80 |  | -1.20 |  | -0.40 |
| Jouini [21] |  | -0.78 |  | -1.20 |  | -0.37 |

^1^Effect sizes were pooled using random-effects mode

**Supplemental Reference**

1.Zhu MW, Tang HC, Zhao L. Determination of trace elements in serum of senile dementia. Modern medical journal, 14(1), 18-19 (1997).

2.Cheng YM, Ge W, Zhang SY et al. A study on serum Ca, Mg, Zn, Al contents in senile dementia. Bulletin of science and technology, 15(6), 467-469 (1999).

3.Alimonti A, Ristori G, Giubilei F et al. Serum chemical elements and oxidative status in Alzheimer's disease, Parkinson disease and multiple sclerosis. Neurotoxicology, 28(3), 450-456 (2007).

4.Cilliler AE, Ozturk S, Ozbakir S. Serum magnesium level and clinical deterioration in Alzheimer's disease. Gerontology, 53(6), 419-422 (2007).

5.Liu, K. The Study of the Association between Trace Element and Senile Dementia/Depressive Disorder. Master’s Thesis, Shandong University, Jinan, China (2008).

6.Gustaw-Rothenberg K, Kowalczuk K, Stryjecka-Zimmer M. Lipids' peroxidation markers in Alzheimer's disease and vascular dementia. Geriatr Gerontol Int, 10(2), 161-166 (2010).

7.Barbagallo M, Belvedere M, Di Bella G, Dominguez LJ. Altered ionized magnesium levels in mild-to-moderate Alzheimer's disease. Magnes Res, 24(3), S115-121 (2011).

8.Singh NK, Banerjee BD, Bala K, Basu M, Chhillar N. Polymorphism in Cytochrome P450 2D6, Glutathione S-Transferases Pi 1 Genes, and Organochlorine Pesticides in Alzheimer Disease: A Case-Control Study in North Indian Population. J Geriatr Psychiatry Neurol, 27(2), 119-127 (2014).

9.Wang, LZ. A case-control study for relevant factors of Alzheimer's disease. Master's Thesis, Inner mongolia medical university, Hohhot, China, 1-40 (2015).

10.Zheng, F. A case-control study on related influence factors of Alzheimer's disease. Master's Thesis, Jilin university, Changchun, China, 1-61 (2015).

11.Koc ER, Ilhan A, Zubeyde A et al. A comparison of hair and serum trace elements in patients with Alzheimer disease and healthy participants. Turk J Med Sci, 45(5), 1034-1039 (2015).

12.Balmus IM, Strungaru SA, Ciobica A et al. Preliminary Data on the Interaction between Some Biometals and Oxidative Stress Status in Mild Cognitive Impairment and Alzheimer's Disease Patients. Oxidative medicine and cellular longevity, 2017, 7156928 (2017).

13.Lemke MR. Plasma magnesium decrease and altered calcium/magnesium ratio in severe dementia of the Alzheimer type. Biol Psychiatry, 37(5), 341-343 (1995).

14.Kurup RK, Kurup PA. Hypothalamic digoxin, hemispheric chemical dominance, and Alzheimer's disease. Int J Neurosci, 113(3), 361-381 (2003).

15.Bostrom F, Hansson O, Gerhardsson L et al. CSF Mg and Ca as diagnostic markers for dementia with Lewy bodies. Neurobiology of aging, 30(8), 1265-1271 (2009).

16.Vural H, Demirin H, Kara Y, Eren I, Delibas N. Alterations of plasma magnesium, copper, zinc, iron and selenium concentrations and some related erythrocyte antioxidant enzyme activities in patients with Alzheimer's disease. Journal of trace elements in medicine and biology: organ of the Society for Minerals and Trace Elements (GMS), 24(3), 169-173 (2010)10

17. Ahmed AS, Elgharabawy RM, Al-Najjar AH. Ameliorating effect of anti-Alzheimer's drugs on the bidirectional association between type 2 diabetes mellitus and Alzheimer's disease. Exp Biol Med (Maywood), 242(13), 1335-1344 (2017).

18.Xu J, Church SJ, Patassini S et al. Plasma metals as potential biomarkers in dementia: a case-control study in patients with sporadic Alzheimer's disease. Biometals: an international journal on the role of metal ions in biology, biochemistry, and medicine, 31(2), 267-276 (2018).

19.Bostrom F, Hansson O, Blennow K et al. Cerebrospinal fluid total tau is associated with shorter survival in dementia with Lewy bodies. Dement Geriatr Cogn Disord, 28(4), 314-319 (2009)

20.Hozumi I, Hasegawa T, Honda A et al. Patterns of levels of biological metals in CSF differ among neurodegenerative diseases. Journal of the neurological sciences, 303(1-2), 95-99 (2011).

21.Jouini N, Saied Z, Ben Sassi S et al. Impacts of Iron Metabolism Dysregulation on Alzheimer's Disease. Journal of Alzheimer's disease : JAD, 80(4), 1439-1450 (2021)
